# Bioelectrical impedance vector analysis for evaluating zinc supplementation in prepubertal and healthy children

**DOI:** 10.3402/fnr.v59.28918

**Published:** 2015-09-29

**Authors:** Márcia Marília Gomes Dantas, Érika Dantas Medeiros Rocha, Naira Josele Neves Brito, Camila Xavier Alves, Mardone Cavalcante França, Maria das Graças Almeida, José Brandão-Neto

**Affiliations:** 1 Department of Internal Medicine, Postgraduate Program in Health Sciences, Federal University of Rio Grande do Norte, Natal, Brazil; 2 Department of Statistics, Federal University of Rio Grande do Norte, Natal, Brazil; 3 Department of Clinical and Toxicological Analyses, Federal University of Rio Grande do Norte, Natal, Brazil; 4 Department of Internal Medicine, Federal University of Rio Grande do Norte, Natal, Brazil

**Keywords:** bioelectrical impedance vector analysis, zinc supplementation, body composition, children

## Abstract

**Background:**

The prevalence of abnormal nutritional status has increased in children and adolescents. Nutritional assessment is important for monitoring the health and nutritional status. Bioelectrical impedance vector analysis (BIVA) combines changes in tissue hydration and structure and body composition that can be assessed.

**Objectives:**

The objective of this study was to use BIVA to evaluate nutritional status in 60 prepubertal children, aged between 8 and 9 years, supplemented with zinc, to detect possible changes in body composition.

**Design:**

We performed a randomized, controlled, triple-blind study. The children were divided into the control group (CG; sorbitol 10%, *n*=29) or the experimental group (EG; 10 mg Zn/day, *n*=31), and the duration of the experiment was 3 months. Anthropometric assessments were performed for all of the children.

**Results:**

The body mass index-for-age increased after oral zinc supplementation in the EG (*p*=0.005). BIVA indicated that the CG demonstrated a tendency for dehydration and decreased soft tissue and the EG demonstrated a tendency for increased soft tissue, primarily the fat-free mass. After analyses of BIVA ellipses, we observed that this method could detect improvements in body composition in healthy children supplemented with zinc.

**Conclusions:**

These results suggest that BIVA could be an auxiliary method for studying a small population undergoing zinc intervention.

The prevalence of nutritional disorders has increased in children and adolescents in developed and developing countries, indicating deficiencies of essential vitamins and minerals (1, 2). This prevalence has resulted in a significant impairment of growth and development in this population (3).

Nutritional assessments are important for monitoring the health and nutritional status of children. Among the many nutritional assessment methods, anthropometry and body composition provide acceptable accuracy with similar discriminative ability when measured by dual-energy X-ray absorptiometry (4, 5).

Methods that accurately assess body composition in children are scarce. International body mass index (BMI)-for-age cutoffs have been proposed to classify overweight and underweight children (6, 7). However, BMI levels among children should be interpreted with caution. Although a high BMI-for-age is a good indicator of excess fat mass (FM), BMI cannot differentiate whether the weight change is due to variations of FM, fat-free mass (FFM), or water (8, 9).

Bioelectrical impedance (BIA) is a user-friendly, non-invasive, low-cost, portable method that can be used to calculate the total body fat in children and adults, and it is considered to be a useful tool for assessing body composition (10–13). However, when using conventional BIA, it is difficult to establish the effect of body weight in prediction equations, and no single equation has been developed to calculate the total body fat in children of different ages. Therefore, an accurate evaluation using the bioelectrical impedance vector analysis (BIVA) is necessary, and the patterns are based only on the electrical properties of the tissues. Combined changes in tissue hydration (resistance component) and structure (reactance component) can be monitored with BIVA. Both components of the impedance vector are considered simultaneously, and body composition can be interpreted (14). The graphical method of resistance and reactance corrected by body length (RXc) is based on analysis of the bivariate impedance vector distribution in a healthy population with specific features (10).

BIVA is a qualitative method and does not provide quantitative measurements of corporal volumes (15–18). BIVA is useful for clinical purposes because of its ability to detect changes in hydration or body composition in children (9). Therefore, BIVA may be helpful in identifying those children who are at risk of pathological changes in body composition, specifically during chronic conditions (i.e. chronic obstructive pulmonary disease, anorexia nervosa, cancer, and chronic renal failure) (14, 19–24).

Supplementary zinc exerts a positive effect on nutritional status through positive weight gain (25). Moreover, zinc is an essential nutrient required for numerous metabolic functions, and its deficiency results in growth retardation, cell-mediated immune dysfunction and cognitive impairment, and decreased protein and nucleic acid synthesis (26).

This study used a BIA vector to evaluate nutritional status in prepubertal healthy and eutrophic children supplemented with zinc to detect changes in body composition status.

## Methods

### Subjects

The participants included 60 healthy and eutrophic prepubertal children aged between 8 and 9 years from three municipal schools in the city of Natal, Brazil. Informed consent was obtained from all of the children and their parents or guardians before data collection. This study was approved by the Onofre Lopes University Hospital Research Ethics Committee of Federal University of Rio Grande do Norte (UFRN) (protocol number 542/11). The subjects were recruited through an advertisement on school noticeboards and meetings with parents. The children were divided into control and experimental groups, and the pairing was performed randomly. The parents and children did not know the status of their experimental group (EG; or oral solution). Only one member of the team controlled the experiments and revealed children's experimental status (control or experimental group) at the time of data collection.

### Inclusion and exclusion criteria

The children were healthy, eutrophic, and at Tanner stage 1 for genital, breast, and pubic hair growth, which was evaluated by a medical doctor. The exclusion criteria were missing or incomplete dietary data; early pubarche, thelarche, or menarche; nutritional disorders; history of disease (neoplasia; diabetes mellitus; liver, kidney, and thyroid disorders; and acute infectious or inflammatory diseases); undergoing surgery; using vitamin or mineral supplements; and children outside the interval −2 to 2+*Z*-score for sex-specific BMI-for-age, weight-for-age, and height-for-age indices, according to the 2006 World Health Organization curves (27).

### Experimental design

This study was a randomized, controlled, triple-blind study and was based on non-probability sampling (convenience sample). The control group (CG) (*n*=29) was supplemented with placebo (10% sorbitol, the same vehicle used to prepare zinc solution) and the EG (*n*=31) was supplemented daily with 10 mg of elemental zinc for 3 months. The CG consisted of 16 males and 13 females, and the EG consisted of 16 males and 15 females. All children underwent anthropometry, BIA measurements, and blood collection to analyze serum zinc at the beginning and end of this study.

### Anthropometry

Anthropometric measurements were performed after an overnight fast. Weight (kg) and height (m) were measured using an electronic balance (Balmak, BK50F, São Paulo, SP, Brazil) and a stadiometer (Stadiometer Professional Sanny, American Medical do Brasil, São Paulo, SP, Brazil), respectively. To measure weight, the child stood on the scale wearing light clothing without shoes. To measure height, the child remained standing without shoes with free head props, heels together, arms extended along the body, and an upright body posture. The heels, buttocks, shoulders, and head touched the wall or vertical surface of the measuring equipment. All measurements were performed by the same examiner to avoid bias (28). New growth curves provided by the World Health Organization for children aged 5 to 19 years were used to classify malnutrition, eutrophic state, and obesity (29). BMI was calculated as the ratio between the body weight (kg) and the square of height in meters (kg/m^2^). BMI was evaluated by the AnthroPlus v1.0.4 program (available at www.who.int/growthref/en/).

### Bioelectrical measurements

Bioimpedance (BIA) is a nutritional assessment method that estimates body composition and therefore nutritional status. Resistance (*R*) and Reactance (*Xc*) are the components of this method that will provide this information. After the child had emptied his/her bladder, BIA was performed with a BIA analyzer (Quantum II, RJL Systems, Comp Corp., Clinton Township, MI, USA). The Houtkooper equation (30) (Table 1), which was validated and currently recommended for use in children, was utilized (31). This technique requires the precise placement of four electrodes (standard tetrapolar placement on the right hand and foot) strictly following the method reported by Lukaski et al. (32). The two components of the whole-body impedance vector were recorded from single representative stable measurements conducted by the same operator.

**Table 1:** Results of body composition obtained in the control (CG, *n*=29) and experimental (EG, *n*=31) groups before and after placebo or oral zinc supplementation in prepubertal and healthy children

	CG-before vs. CG-after	CG-before vs. EG-before	CG-before vs. EG-after	CG-after vs. EG-before	CG-after vs. EG-after	EG-before vs. EG-after
BMI-for-age (kg/m^2^)						
Mean difference	−0.1966	−0.08854	−0.4982	0.1080	−0.3017	−0.4097
95% CI of difference	−1.444 to 1.051	−1.316 to 1.139	−1.726 to 0.729	−1.119 to 1.335	−1.529 to 0.925	−1.617 to 0.797
Significance	NS	NS	NS	NS	NS	*p*=0.005
*R/H* (Ohm/m)						
Mean difference	−1.098	1.905	10.30	3.002	11.40	8.398
95% CI of difference	−53.10 to 50.90	−49.25 to 53.06	−40.85 to 61.45	−48.15 to 54.15	−39.75 to 62.55	−41.89 to 58.69
Significance	NS	NS	NS	NS	NS	NS
*Xc/H* (Ohm/m)						
Mean difference	−0.5828	1.163	0.7157	1.746	1.298	−0.4474
95% CI of difference	−5.614 to 4.448	−3.786 to 6.112	−4.234 to 5.665	−3.203 to 6.695	−3.651 to 6.248	−5.313 to 4.419
Significance	NS	NS	NS	NS	NS	NS

BMI, body mass index; *R*, resistance; *H*, height; *Xc*, reactance. NS, not significant (*p*>0.05) and significant (*p*=0.005), using Tukey's multiple comparisons test.

### Resistance (R)

The *R* is the opposition to flow of an alternating current through intra- and extracellular ionic solutions representing the real portion of the impedance (*Z*). Lean tissues are excellent conductors of electric current due to the large amount of water and electrolytes, that is, they have low *R* to the passage of electric current. On the contrary, fat, bone, and skin constitute a means of low conductivity, and therefore have high *R*.

### Reactance (Xc)

The *Xc* is the capacitance produced by tissue interfaces and cell membranes representing the imaginary portion of the *Z* across tissues (33). *Xc* means the opposition to an electric current caused by the capacitance (property of storing energy in the form of an electrostatic field). The combination of these two values provides information about total body water, FFM, and FM.

### BIVA

The BIVA plots direct measurements of the vectors *R* and *Xc* from the impedance analyzer (RXc graph). According to the RXc graph, impedance measurements standardized by the height of the subject are represented as bivariate vectors with their confidence and tolerance intervals, which are ellipses in the RXc plane. These vectors do not depend on equations (34). The BIVA according to the RXc method is a good indicator of clinical outcomes and for clinical research studies aiming to identify disorders in body composition (35, 36). To investigate the differences between groups, we calculated and plotted the 95% confidence intervals for the average bivariate vector impedance for each group. Furthermore, we calculated and plotted the tolerance ranges of 95, 75, and 50% for the children, which were divided according to the group and time of the study. Healthy Italian children were used as a reference population to compare bioelectrical data (36).

### Oral zinc supplementation

The EG was supplemented with 10 mg Zn per day for 3 months in the form of zinc sulfate heptahydrate (ZnSO_4_·7H_2_O; Merck, Darmstadt, Germany). Oral zinc solution (152.97 µmol Zn/day) was prepared at the Pharmacotechnical Laboratory of the Department of Pharmacy, UFRN. Each drop contained 1 mg of elemental zinc. The CG received an oral placebo as sorbitol 10%. These solutions were added to milk or juice every morning at breakfast. Zinc supplementation was monitored every 2 weeks during home visits by the same observer.

### Materials

Vacuette Z serum clot activator tubes (Greiner Bio-One, Monroe, NC, USA) were used for biochemical analyses. Becton Dickinson tubes (Trace Element, Serum, Franklin Lakes, NJ, USA) were used for zinc analyses. Polypropylene plastic syringes were purchased from BD (Hercules, CA, USA), and plastic tips and tubes (metal-free) were purchased from BioRad Laboratories (Hercules, CA, USA). Zinc sulfate heptahydrate (ZnSO_4_·7H_2_O) and Titrisol zinc standard were purchased from Merck (Darmstadt, Germany).

### Laboratory procedures

All blood samples were collected into the appropriate tubes, and the procedures related to the handling of zinc samples were performed in accordance with international standards (37). After sample collection, the laboratory procedures were performed at the Multidisciplinary Laboratory of Chronic Degenerative Diseases. The blood samples were placed in trace metal-free tubes without anticoagulants and remained in a stainless steel incubator (FANEM 502, São Paulo, SP, Brazil) for 120 min until clot formation occurred. A 500 mL volume of serum was collected with plastic, trace-metal-free pipettes and was transferred to plastic tubes containing 2,000 μL ultra-pure water (Milli-Q Plus, Millipore, Billerica, MA, USA) to dilute the serum (1:4) for zinc analyses. The samples were stored at −80°C for subsequent analyses (Ultralow Freezer, Nuaire, MN, USA). The serum zinc samples were analyzed in triplicates within the same assay by atomic absorption spectrophotometry (SpectrAA-240FS, Varian, Mulgrave, Victoria, Australia) according to the manufacturer's instructions. Zinc sensitivity was 0.01 μg/mL. The intra-assay coefficient of variation was 2.37%. The normal reference range was 0.7–1.2 μg/mL. A standard zinc solution (1,000 mg/mL) was obtained by diluting a Titrisol zinc standard in ultra-pure water. The wavelength was 213.9 nm. The lamp current was 10 mA, and all other procedures, such as calibrations and measurements, were performed according to the manufacturer's instructions.

### Statistical analyses

The D'Agostino–Pearson omnibus normality test was used to analyze the normality of all study data. Paired Student's *t*-test was used to compare the data obtained in the control and experimental groups. The Wilcoxon matched-pairs signed rank test was used to complement the paired, non-parametric test. All comparisons were considered to be significant at the 5% significance level. All analyses and figures were performed and created with the GraphPad Prism 6.0 software (GraphPad Software, Inc., San Diego, CA, USA). The BIVA statistical analyses were performed using BIVA software 2003. To determine the group differences, the bivariate 95% confidence interval for the mean impedance vector was calculated and plotted using the bivariate normal distribution of *R* divided by height (*R/H*) and Xc divided by height (*Xc/H*). The unpaired, two-sample Hotelling's *T* ^2^ test for vector analysis was performed. Separate 95% confidence ellipses indicated a significant difference between the mean vector positions on the RXc plane (equivalent to a significant two-sample Hotelling's *T* ^2^ test, *p*<0.05). The paired, one-sample Hotelling's *T* ^2^ test was performed to determine if the changes in the mean group vectors (measured at the first and second time points) were significantly different from zero (null vector). A 95% confidence ellipse excluding the null vector indicated a significant vector displacement (31). These tests are a multivariate extension of Student's *t*-test. To determine whether the results were true for the population studied, we used a sample size calculation for the comparison of two means (paired samples) as follows: *n*=(*Zα+Zβ*)^2^.*σ*^2^_D_/*δ*^2^, where *α*=0.05, *P* (test power)=0.90, *β*=2(1−*P*)=0.20, *σ*^2^D (standard deviation of the difference)=0.105043, and *δ*>−0.09 (maximum permissible difference).

## Results

This study was conducted with 29 children in the CG and 31 children in the EG who underwent anthropometric and bioelectrical assessments. The sample size (60 children) was adequate for the conclusions in this study. For any value, *δ*≥−0.09 (i.e. the minimum sample size required was 15 patients). According to the BMI-for-age classification, all children were healthy during the 3-month study (Table 1).

Assuming a bivariate normal distribution of *R/H* and *Xc/H*, we calculated the bivariate 95% confidence limits for the mean impedance vectors of the different classification groups (i.e. the ellipse within the two-dimensional mean vector falls with a 95% probability). The ‘RXc mean graph’ was the average of *R/H* and *Xc/H* recorded the groups (38).

**Fig. 1:**
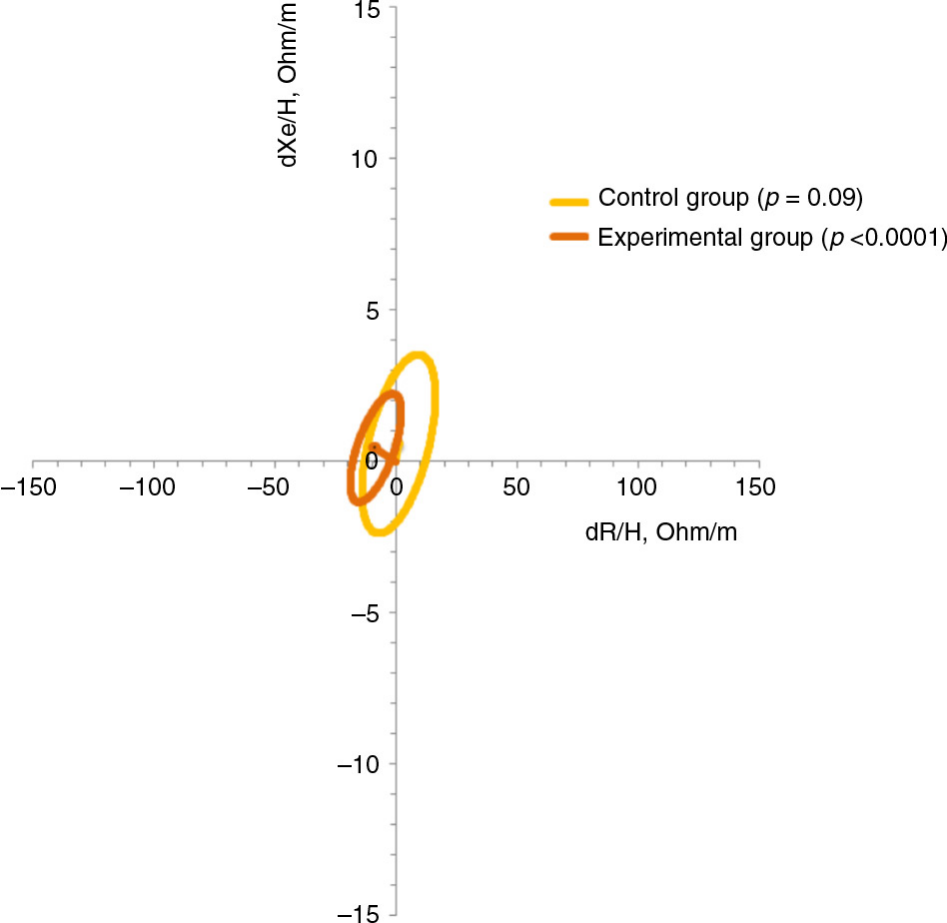
The 95% confidence ellipses of impedance vectors measured by the difference between before and after placebo (control group) and before and after zinc supplementation (experimental group).

The mean vectors for the CG and EG were plotted before and after placebo and supplementation. The 95% confidence ellipses of the three mean vectors of each group overlapped, which indicates that the position between the vectors at the CG, EG, and reference population (healthy Italian children) were not significantly different in the RXc plane. The paired one-sample Hotelling's *T* ^2^ test indicated a difference in the mean vectors between the first measurement (before placebo or supplementation) and the second measurement (after placebo or supplementation). The EG had increased in the soft tissue (primarily FFM) body composition after oral zinc supplementation compared with before supplementation (*p*<0.0001). However, the *p* value of the paired one-sample Hotelling's *T* ^2^ test for the CG was 0.9 (Fig. 1).

**Fig. 2:**
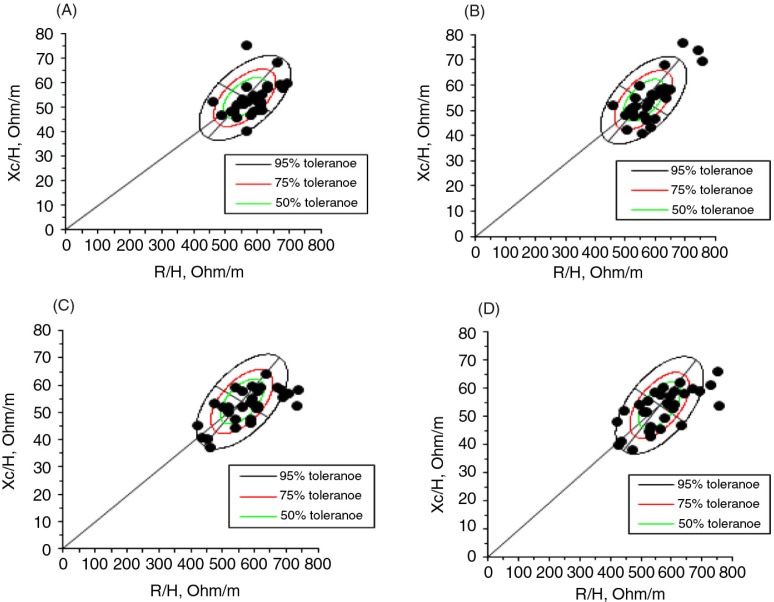
Distribution of impedance vectors with the 50, 75, and 95% tolerance ellipses for (A) the control group before placebo, (B) the control group after placebo, (C) the experimental group before zinc supplementation, and (D) the experimental group after zinc supplementation. R/H, resistance/length; Xc/H, reactance/length.

We drew bivariate 50, 75, and 95% tolerance intervals of the impedance vector in the reference population. We then plotted the distribution of individual vectors on the reference ellipses, which allowed for the comparison of the bivariate, intersubject impedance variability. These ellipses were separated by groups before and after placebo or zinc supplementation versus the reference population (healthy Italian children) (Fig. 2).

The distribution of individual vectors reflected the heterogeneity of hydration status in eutrophic children ranging from severe dehydration (vectors beyond the upper pole of the 95% tolerance ellipse) to pre-edema fluid overload (vectors close to the lower pole of the 75% tolerance ellipse) in both groups. The CG demonstrated a tendency for dehydration and decreased soft tissue. The EG demonstrated a tendency for increased soft tissue (primarily the FFM).

**Fig. 3:**
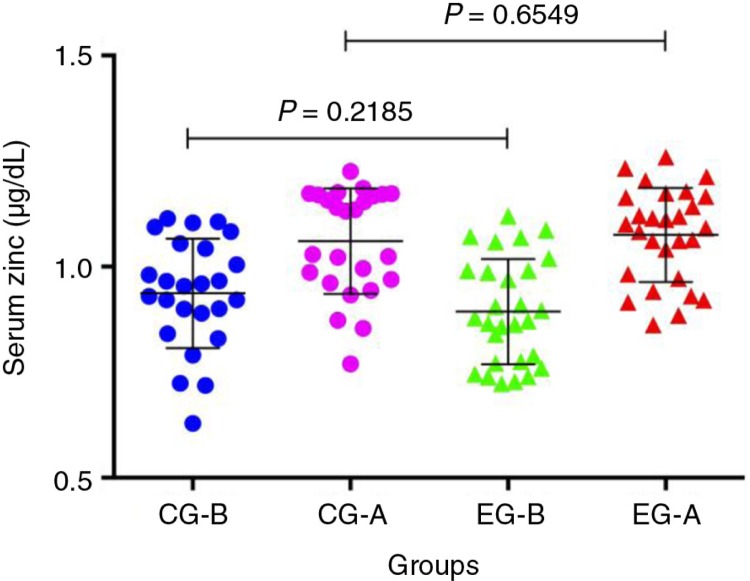
Serum zinc levels in the control group before placebo (CG-B) and after placebo (CG-A) and in the experimental group before zinc supplementation (EG-B) and after zinc supplementation (EG-A).

The serum zinc levels were not significantly different between the groups (Fig. 3). However, zinc intake plus zinc supplementation was significantly different in the EG when compared with a CG (*p*<0.0001). Moreover, the tolerable upper zinc intake level did not exceed the expected values of 23 mg Zn per day for children in the EG. There were no side effects associated with the 10 mg Zn per day.

## Discussion

In this study on 60 healthy and eutrophic children, we compared the effect of zinc supplementation on nutritional status as evaluated by BIVA for assessing body composition. We obtained the tolerance intervals of ellipses in children aged between 8 and 9 years. BIVA is a clinically useful method and can be used for routine monitoring of variations in body fluids and nutritional status in children.

The length of the vector indicates hydration status, and a left/right shift of the vector indicates soft tissue mass (35). In clinical validation studies in adults, vectors falling out of the 75% tolerance ellipse indicate abnormal tissue impedance. An upper pole or lower pole displacement of the vectors parallel to the major axis of tolerance ellipses indicates tissue dehydration or hyper-hydration, respectively. Extremes of soft tissue mass, such as in obese people and athletes, or lean and wasting conditions are associated with a vector displacement to the left or to the right, respectively, along the minor axis of tolerance ellipses (36, 37, 39).

To date, longitudinal data regarding the use of BIA vector for evaluating children after zinc supplementation have not been reported in the literature. We found positive changes in the body composition with zinc supplementation. BIVA in healthy neonates provides good results for obtaining nutritional status and body fluids, and their clinical state could be predicted depending on their location within the quadrants of the graphic ellipses (38). BIVA has been used for detection, monitoring, and controlling hydration and nutritional status using vector displacement for feedback among patients with Alzheimer's disease (40), cachexia (41), stable and non-stable heart failure patients (42), critically ill and cardiorenal patients (43), hemodialysis patients (44), and cancer patients (28, 45). However, there are no references in the literature regarding zinc intervention and BIVA to compare with our study. Therefore, we did not have a reference method or gold standard for the estimation of body composition, and the results should be interpreted with caution.

Concerning anthropometric assessment, the BMI-for-age increased after oral zinc supplementation in the EG. This physiological effect is expected, even in healthy children (46). All children were classified as eutrophic throughout the study using the BMI-for-age. Some studies have demonstrated that BIVA has an advantage compared with other methods because BIVA is a good identifier of individual vectors indicating changes in tissue hydration and body structure in subjects from any BMI class. Guida et al. (9) reported a BMI-specific difference in vector position in an 8-year-old age group with progressive vector shortening in groups with increasing BMI class. We used BIVA to observe whether there were changes in body composition with oral zinc supplementation. We suggest that this supplementation changed the body composition of children because the *p* value in the EG was<0.0001 (calculated by the BIVA software).

### Limitations

The main limitation of this study was its relatively small sample size. Nevertheless, the rate of compliance was satisfactory. As our sample was not probabilistic, other studies with a more representative population are required. Moreover, this was an innovative study that used a novel methodological approach, and there were no previous references in the scientific literature using BIVA to assess a nutritional intervention.

## Conclusions

Following the analyses of ellipses by BIVA, we found that this method may detect improvements in body composition (primarily the FFM) in healthy children supplemented with zinc. This finding suggests that BIVA can be used to study a small population undergoing zinc (or other micronutrients) intervention. However, longitudinal data are required to investigate vector migration during zinc supplementation. Monitoring the vector displacement trajectory toward the reference target vector position may represent useful feedback during nutritional therapy. Other studies are required to confirm our results. Currently, BIVA is still not considered to be a gold standard.

## References

[1] Ogden CL, Carroll MD Prevalence of obesity among children and adolescents: United States, trends 1963–1965 through 2007–2008. Health E-Stat 2010.

[2] Black RE, Allen LH, Bhutta ZA, Caulfield LE, de Onis M, Ezzati M (2008). Maternal and child undernutrition: global and regional exposures and health consequences. Lancet.

[3] Washi SA, Ageib MB (2010). Poor diet quality and food habits are related to impaired nutritional status in 13- to 18-year-old adolescents in Jeddah. Nutr Res.

[4] Wells JC, Fewtrell MS (2008). Is body composition important for paediatricians?. Arch Dis Child.

[5] Krachler B, Völgyi E, Savonen K, Tylavsky FA, Alén M, Cheng S (2013). BMI and an anthropometry-based estimate of fat mass percentage are both valid discriminators of cardiometabolic risk: a comparison with DXA and bioimpedance. J Obes.

[6] Cole TJ, Bellizzi MC, Flegal KM, Dietz WH (2000). Establishing a standard definition for child overweight and obesity worldwide: international survey. BMJ.

[7] Cole TJ, Flegal KM, Nicholls D, Jackson AA (2007). Body mass index cut offs to define thinness in children and adolescents: international survey. BMJ.

[8] Freedman DS, Wang J, Maynard LM, Thornton JC, Mei Z, Pierson RN (2005). Relation of BMI to fat and fat-free mass among children and adolescents. Int J Obes.

[9] Guida B, Pietrobelli A, Trio R, Laccetti R, Falconi C, Perrino NR (2008). Body mass index and bioelectrical vector distribution in 8-year-old children. Nutr Metab Cardiovasc Dis.

[10] L'Abée C, Poorts-Borger PH, Gorter EH, Piccoli A, Stolk RP, Sauer PJ (2010). The bioelectrical impedance vector migration in healthy infants. Clin Nutr.

[11] Savino F, Grasso G, Cresi F, Oggero R, Silvestro L (2003). Bioelectrical impedance vector distribution in the first year of life. Nutrition.

[12] Kushner RF, Gudivaka R, Schoeller DA (1996). Clinical characteristics influencing bioelectrical impedance analysis measurements. Am J Clin Nutr.

[13] Kyle UG, Bosaeus I, De Lorenzo AD, Deurenberg P, Elia M, Manuel Gómez J (2004). Bioelectrical impedance analysis-part II: utilization in clinical practice. Clin Nutr.

[14] Chertow GM, Lazarus JM, Lew NL, Ma L, Lowrie EG (1997). Bioimpedance norms for the hemodialysis population. Kidney Int.

[15] Victor RP (2012). Handbook of anthropometry: physical measures of human form in health and disease.

[16] Norman K, Pirlich M, Sorensen J, Christensen P, Kemps M, Schütz T (2009). Bioimpedance vector analysis as a measure of muscle function. Clin Nutr.

[17] Ronco C, Crepaldi C, Cruz DN (2009). Peritoneal dialysis: from basic concepts to clinical excellence.

[18] Roa LM, Naranjo D, Reina-Tosina J, Lara A, Milán JA, Estudillo MA, Azar AT (2013). Applications of bioimpedance to end stage renal disease (ESDR). Modeling and control of dialysis systems.

[19] Piccoli A (1998). Identification of operational clues to dry weight prescription in hemodialysis using bioimpedance vector analysis. The Italian hemodialysis-bioelectrical impedance analysis (HD-BIA) study group. Kidney Int.

[20] Guida B, De Nicola L, Trio R, Pecoraro P, Iodice C, Memoli B (2000). Comparison of vector and conventional bioelectrical impedance analysis in the optimal dry weight prescription in hemodialysis. Am J Nephrol.

[21] Pillon L, Piccoli A, Lowrie EG, Lazarus JM, Chertow GM (2004). Vector length as a proxy for the adequacy of ultrafiltration in hemodialysis. Kidney Int.

[22] Walter-Kroker A, Kroker A, Matticucci-Guehlke M, Glaab T (2011). A practical guide to bioelectrical impedance analysis using the example of chronic obstructive pulmonary disease. Nutr J.

[23] Piccoli A, Codognotto M, Di Pascoli L, Boffo G, Caregaro L (2005). Body mass index and agreement between bioimpedance and anthropometry estimates of body compartments in anorexia nervosa. JPEN-Parenter Enter.

[24] Brooks ER, Fatallah-Shaykh SA, Langman CM, Wolf KM, Price HE (2008). Bioelectric impedance predicts total body water, blood pressure, and heart rate during hemodialysis in children and adolescents. J Renal Nutr.

[25] Prasad AS (2013). Discovery of human zinc deficiency: its impact on human health and disease. Adv Nutr.

[26] Consolo LZ, Melnikov P, Cônsolo FZ, Nascimento VA, Pontes JC (2013). Zinc supplementation in children and adolescents with acute leukemia. Eur J Clin Nutr.

[27] WHO Multicentre Growth Reference Study Group (2006). WHO child growth standards: length/height-for-age, weight-for-age, weight-for-length, weight-for-height and body mass index-for-age: methods and development.

[28] Malecka-Massalska T, Smolen A, Zubrzycki J, Lupa-Zatwarnicka K, Morshed K (2012). Bioimpedance vector pattern in head and neck squamous cell carcinoma. J Physiol Pharmacol.

[29] Brasil. Ministério da Saúde (2004). Vigilância Alimentar e Nutricional – SISVAN: orientações básicas para a coleta, processamento, análise dos dados e informação em serviços de saúde.

[30] Houtkooper LB, Going SB, Lohman TG, Roche AF, Van Loan M (1992). Bioelectrical impedance estimation of fat-free body mass in children and youth: a cross-validation study. J Appl Physiol.

[31] Barbosa-Silva MC, Barros AJ (2005). Bioelectrical impedance analysis in clinical practice: a new perspective on its use beyond body composition equations. Curr Opin Clin Nutr Metab Care.

[32] Lukaski HC, Bolonchuk WW, Hall CB, Siders WA (1986). Validation of tetrapolar bioelectrical impedance method to assess human body composition. J Appl Physiol.

[33] Heyward VH, Wagner DR (2004). Applied body composition assessment.

[34] Kyle UG, Bosaeus I, De Lorenzo AD, Deurenberg P, Elia M, Gómez JM (2004). Bioelectrical impedance analysis – part I: review of principles and methods. Clin Nutr.

[35] Piccoli A, for the Italian CAPD-BIA study group (2004). Bioelectric impedance vector distribution in peritoneal dialysis patients with different hydration status. Kidney Int.

[36] Piccoli A, Pastori G (2002). BIVA software.

[37] Lowe N, Fekete K, Decsi T (2009). Methods of assessment of zinc status in humans: a systematic review. Am J Clin Nutr.

[38] Margutti AV, Monteiro JP, Camelo JS (2010). Reference distribution of the bioelectrical impedance vector in healthy term newborns. Brit J Nutr.

[39] Tanabe RF (2010). Valores de referência do vetor de bioimpedância elétrica corporal total em lactentes e pré-escolares [dissertação].

[40] Buffa R, Mereu RM, Putzu PF, Floris G, Marini E (2010). Bioelectrical impedance vector analysis detects low body cell mass and dehydration in patients with Alzheimer's disease. J Nutr Health Aging.

[41] Castillo-Martínez L, Colín-Ramírez E, Orea-Tejeda A, González Islas DG, Rodríguez García WD, Santillán Díaz C (2012). Cachexia assessed by bioimpedance vector analysis as a prognostic indicator in chronic stable heart failure patients. Nutrition.

[42] Gastelurrutia P, Nescolarde L, Rosell-Ferrer J, Domingo M, Ribas N, Bayes-Genis A (2011). Bioelectrical impedance vector analysis (BIVA) in stable and non-stable heart failure patients: a pilot study. Int J Cardiol.

[43] Peacock WF (2010). Use of bioimpedance vector analysis in critically ill and cardiorenal patients. Contrib Nephrol.

[44] Nescolarde L, Piccoli A, Román A, Núñez A, Morales R, Tamayo J (2004). Bioelectrical impedance vector analysis in haemodialysis patients: relation between oedema and mortality. Physiol Meas.

[45] Toso S, Piccoli A, Gusella M, Menon D, Bononi A, Crepaldi G (2000). Altered tissue electric properties in lung cancer patients as detected by bioelectric impedance vector analysis. Nutrition.

[46] Leite LD, Rocha EDM, Almeida MG, Rezende AA, Silva CAB, França MC (2009). Sensitivity of zinc kinetics and nutritional assessment of children submitted to venous zinc tolerance test. J Am Coll Nutr.

